# Isolation and Characterization of Porcine Epidemic Diarrhea Virus G2c Strains Circulating in China from 2021 to 2024

**DOI:** 10.3390/vetsci12050444

**Published:** 2025-05-06

**Authors:** Xi Lu, Chen Chen, Zixuan Wang, Anding Zhang

**Affiliations:** 1 State Key Laboratory of Agricultural Microbiology, Hubei Hongshan Laboratory, College of Veterinary Medicine, Huazhong Agricultural University, Wuhan 430070, China; lucylook5@webmail.hzau.edu.cn (X.L.); chen110@webmail.hzau.edu.cn (C.C.); kakawzx@163.com (Z.W.); 2 Key Laboratory of Preventive Veterinary Medicine in Hubei Province, The Cooperative Innovation Center for Sustainable Pig Production, Wuhan 430070, China; 3 Shenzhen Kingkey Smart Agriculture Times Co., Ltd., Shenzhen 518000, China; 4 Key Laboratory of Development of Veterinary Diagnostic Products, Ministry of Agriculture of the People’s Republic of China, Wuhan 430070, China; 5 International Research Center for Animal Disease, Ministry of Science and Technology of the People’s Republic of China, Wuhan 430070, China

**Keywords:** porcine epidemic diarrhea virus (PEDV), virus isolation, antigenic variation, vaccine efficacy, G2c genotype

## Abstract

Porcine epidemic diarrhea virus (PEDV) is a major pathogen responsible for viral diarrhea in pigs, causing particularly high mortality in neonatal piglets. In recent years, genetic variations in PEDV have resulted in alterations in both its virulence and antigenicity, leading to a reduced efficacy of existing vaccines. In this study, diarrheal samples were collected from four commercial pig farms in the Hubei, Guangxi, and Jiangxi provinces, China, which experienced vaccine failure. RT-qPCR confirmed PEDV infection, and three PEDV strains, 2021-HBMC, 2024-JXYX, and 2024-JXNC, were successfully isolated. Sequence analysis and phylogenetic tree construction classified these strains into the G2c genotype, the predominant subtype in China. The neutralization assays revealed a significant reduction in the neutralizing titers of these strains against the immune serum compared with the AJ1102 reference strain. Further amino acid sequence analysis of the spike (S) protein identified several mutations in key neutralizing epitopes compared with the AJ1102 strain, including S27L, E57A, N139D, M214T, and P229L in the S-NTD epitope; A520S, F539L, K566N, D569E, G612V, P634S, E636V/K in the COE epitope; and Y1376H in the 2C10 epitope, along with several deletions at N-glycosylation sites (347NSSD and 510NITV). Additionally, whole-genome sequencing and recombination analysis indicated that the 2021-HBMC strain may have resulted from a recombination event. The findings of this study underscore the challenge posed by the continuous genetic evolution of PEDV to vaccine efficacy and provide valuable insights for future vaccine development and control strategies.

## Simple Summary

This study successfully isolated three Porcine epidemic diarrhea virus strains from immune-failure pig farms in China, designated 2021-HBMC, 2024-JXNC, and 2024-JXYX. All three strains were classified as G2c, the most prevalent genotype in China. They exhibit several mutations in the major neutralizing epitopes of the S protein compared to the AJ1102 strain, along with deletions in some N-glycosylation sites, which may explain the reduced neutralizing activity of the vaccine serum against them. These findings highlight the widespread prevalence of the G2c genotype in China and suggest that current vaccines are less effective against it. This underscores the current PEDV epidemic situation and informs future vaccine development and control strategies.

## 1. Introduction

Porcine epidemic diarrhea virus (PEDV) is a primary pathogen responsible for viral diarrhea in pigs, causing symptoms such as watery diarrhea, vomiting, and severe dehydration [1,2]. PEDV can infect pigs of various breeds and ages, with the greatest threat to neonatal piglets, where morbidity and mortality rates can reach up to 100%, leading to substantial economic losses to the global swine industry [3,4,5]. Since 2010, PEDV has caused numerous outbreaks worldwide, particularly in Asia and North America, with a notable increase in epidemic intensity and severity, making it one of the major challenges facing the swine industry [6,7,8].

PEDV belongs to the *Coronaviridae* family and *Alphacoronavirus* genus and has a single-stranded positive-sense RNA genome, characterized by a high mutation rate and a propensity for recombination [9]. Based on the genetic variability of the highly mutable spike (S) gene, PEDV is classified into two major genotypes, G1 and G2, which are further subdivided into G1a, G1b, G2a, and G2b [10]. PEDV was first discovered in the United Kingdom in 1977 and was introduced into China in the 1980s, initially causing only regional outbreaks [11,12]. However, in 2010, a G2 variant emerged that was distinct from the classic G1 strain, exhibiting increased virulence, which led to higher morbidity and mortality in piglets and caused severe economic losses to the swine industry in Asia [13,14,15]. In 2013, the highly virulent G2 strain emerged in the United States, rapidly spreading across multiple states and gradually evolving into a global epidemic [16,17]. Subsequently, in 2014, an S-INDEL strain, arising from recombination between G1 and G2 strains, emerged, showing higher homology to the G1 strain in the S protein but relatively lower virulence [16,17,18].

Vaccination is one of the primary strategies for controlling PEDV; however, the virus’ complex and rapid genetic variation in recent years has led to changes in its pathogenicity, tissue tropism, and antigenicity, challenging the effectiveness of current vaccines [19,20,21]. Between 2010 and 2020, PEDV infections were reported in nearly all provinces of China, with the G2a and G2b strains predominantly circulating between 2010 and 2016 [22,23]. A modified live vaccine developed from the G2b strain AJ1102 was released in late 2017 and widely adopted, helping to curb the spread of PEDV to some extent [24,25]. However, after 2020, despite widespread vaccination in most pig farms, several studies reported PEDV outbreaks, with the strains being identified as a new subtype G2c [26,27,28]. These studies suggest that the incidence of G2c strains has increased in recent years, and existing vaccines may not effectively prevent G2c PEDV infections. Therefore, ongoing monitoring of PEDV, including strain epidemiology and genetic variation, is essential for the development of more effective vaccines and the formulation of evidence-based control strategies.

In this study, a total of 17 PEDV-positive samples (including feces and intestinal tissue) were collected from commercial pig farms in the Hubei, Guangxi, and Jiangxi provinces of China, where PEDV outbreaks had occurred. RT-qPCR testing confirmed that PEDV was the primary cause of diarrhea in these farms, and three representative PEDV strains were successfully isolated. Sequence analysis and phylogenetic tree construction classified these strains as G2c. Further neutralization tests and analysis of the S protein amino acid sequences identified potential reasons for the vaccination failures. This study examined the genetic evolution of and variation in these G2c strains compared to representative strains from Asia, providing valuable insights into the current PEDV epidemic situation and informing future vaccine development and control strategies.

## 2. Materials and Methods

### 2.1. Sample Collection and Processing

From 2021 to 2024, a total of 17 PEDV-positive samples were conveniently collected from 4 commercial farms with PEDV outbreaks in the Hubei (n = 1), Guangxi (n = 1), and Jiangxi (n = 2) provinces, each housing 2000–3000 sows. Specifically, 1 PEDV-positive sample was collected from a farm in Hubei Province, 6 from a farm in Guangxi Province, and 5 each from two farms in Jiangxi Province. The piglets experienced watery diarrhea and dehydration, leading to mortality. The collected samples were diluted with an equal volume of Dulbecco’s Modified Eagle Medium (DMEM) (Gibco, Grand Island, NE, USA) and homogenized using a multi-sample tissue grinder (Lichen-BX, Shanghai, China). The homogenate was then centrifuged at 12,000 rpm for 5 min to separate the supernatant, which was subsequently filtered through a 0.22 μm filter (Sigma-Aldrich, Burlington, MA, USA) to obtain the viral solution.

### 2.2. Viral RNA Isolation and RT-qPCR Detection

Following the manufacturer’s instructions, 200 μL of the viral solution was processed for nucleic acid extraction using the Singuway Nucleic Acid Extraction Kit and Extraction Instrument (Singuway, Shenzhen, China). Pathogen detection was performed via a quadruplex RT-qPCR assay developed in our laboratory (unpublished data), which detects the viral loads of PEDV, TGEV, PoRV, and PDCoV in the samples via a probe-based method. Specifically, specific primers, probes for PEDV, TGEV, PoRV, and PDCoV, and a one-step RT-qPCR enzyme (cwbio, Taizhou, China) were prepared in a 15 μL reaction mixture. Then, 5 μL of the sample RNA was added to the mixture, and RT-qPCR was performed using a multi-channel qPCR detection device (Tianlong, Xi’an, China).

### 2.3. PEDV Isolation

The filtered viral solution was supplemented with trypsin (Sigma-Aldrich, Burlington, MA, USA) to achieve a final concentration of 10 μg/mL and then inoculated onto Vero cells, which had been washed three times with PBS. The cells were incubated for 2 h at 37 °C in a 5% CO_2_ incubator. After incubation, the inoculum was discarded, and the cells were replenished with DMEM containing 10 μg/mL trypsin for further incubation at 37 °C with 5% CO_2_ for 48 to 72 h. The cells with noticeable cytopathic effects were collected for three freeze–thaw cycles and then passaged into Vero cell monolayers for four consecutive generations. After the fifth passage, viral RNA was extracted, and the viral load was quantified using RT-qPCR.

### 2.4. Immunofluorescence Assay

The 2021-HBMC, 2024-JXNC, and 2024-JXYX strains were inoculated onto Vero cell monolayers and cultured for 24 h. Upon the appearance of significant cytopathic effects, the cells were fixed with a solution of 50% acetone and 50% anhydrous ethanol for 15 min. The cells were then washed three times with PBS. Next, a PEDV N monoclonal antibody (prepared in our laboratory) was added and incubated at 37 °C for 1 h. After three washes with PBS, the cells were incubated with DyLight488-conjugated goat anti-mouse IgG (Abbkine, Wuhan, China) at 37 °C for 1 h. Following three PBS washes, the cells were stained with DAPI (Beyotime, Shanghai, China) for 10 min. After another round of PBS washes, fluorescence was observed under a fluorescence microscope.

### 2.5. Virus TCID_50_ Assay and Serum Neutralization Test

The PEDV strain, capable of inducing cytopathic effects (CPEs) on Vero cells, was used for the TCID_50_ assay. The virus was diluted tenfold in DMEM supplemented with 10 μg/mL trypsin and subsequently added to Vero cell monolayers, which had been washed three times with PBS. TCID_50_ was determined using the Reed–Muench method.

For the serum neutralization test, serum was collected from sows immunized with a commercial vaccine (based on the AJ1102 strain) from a pig farm in Hubei. The serum was inactivated by incubation at 56 °C for 30 min and then diluted twofold in DMEM containing 10 μg/mL trypsin. Equal volumes of the diluted serum were mixed with 200 TCID_50_ of the virus and incubated for 1 h at 37 °C. The serum–virus mixture was then added to Vero cell monolayers, which had been washed twice with PBS, and incubated for 2 h at 37 °C in a 5% CO_2_ incubator. After discarding the mixture, the cells were washed twice with DMEM containing 10 μg/mL trypsin and cultured for 24 h under the same CO_2_ incubation conditions. The neutralizing titer of the serum was determined based on the highest serum dilution that inhibited 50% of the CPE.

### 2.6. Sequencing, Multiple-Sequence Alignment, and Phylogenetic Analysis

RNA was extracted from PEDV-positive samples with high viral concentrations. cDNA was synthesized using random primers and reverse transcriptase (Vazyme, Nanjing, China). The S gene was amplified using the three primer pairs listed in Table S1 and Vazyme Phanta Max Super-Fidelity DNA Polymerase (Vazyme, Nanjing, China). The amplified products were sent to Qingke Biotechnology (Wuhan, China) for sequencing and assembly, with the 2021-HBMC sample undergoing whole-genome sequencing. The obtained S gene sequences were aligned with the CV777 and AJ1102 vaccine strains, as well as 2180 other S gene sequences available in the NCBI database (Table S2) for sequence comparison and phylogenetic analysis. The genome sequences of the 2021-HBMC sample and other reference genomes were also aligned and analyzed phylogenetically with 290 reference genomes (Table S3). Sequence alignment was performed using MAFFT 7.526 with default settings. Phylogenetic trees for the S gene and the entire genome were constructed using the Neighbor-Joining method with 1000 bootstrap replicates, implemented in MEGA 7.0 software. The resulting trees were visualized using the Chiplot online platform [29].

### 2.7. Amino Acid Sequence Analysis of the Spike Protein

To examine the genetic characteristics of the PEDV strains, the spike (S) protein sequences of the isolated strains were aligned with representative strains from various genotypes using MEGA 7.0 software. The variations in the neutralizing epitopes of the S protein were analyzed. Sequence editing and refinement were carried out using Jalview 2.11.4.1. Additionally, online tools were employed to predict changes in N-glycosylation sites (https://services.healthtech.dtu.dk/services/NetNGlyc-1.0/) (accessed on 3 February 2025), with N-glycosylation sites scoring “++” or higher considered highly specific [30].

### 2.8. Viral Recombination Analysis

Recombination events in the isolated viruses were assessed using RDP v4.101 software, employing seven methods (RDP, GENECONV, BootScan, MaxChi, Chimaera, SiScan, 3Seq) set to their default configurations. A recombination event was considered reliable if at least six methods provided a positive result with a *p*-value of less than 10^−6^.

## 3. Results

### 3.1. Viral Isolation and Identification

**Figure 1:**
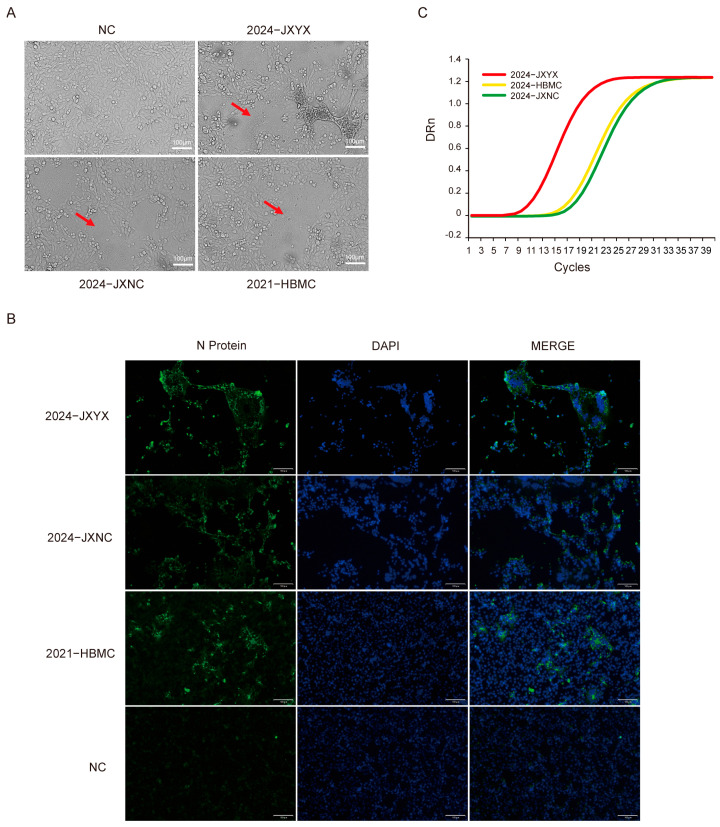
Isolation and characterization of the PEDV strains 2021-HBMC, 2024-JXNC and 2024-JXYX. (**A**) Vero cells were infected with the 2021-HBMC, 2024-JXNC, or 2024-JXYX strain for 24 h; the cytopathic effect was observed using a microscope, and the red arrows indicate the syncytium area. (**B**) Vero cells were infected with the 2021-HBMC, 2024-JXNC, or 2024-JXYX strain for 24 h and were detected using an immunofluorescence assay. (**C**) Detection of PEDV-N gene expression levels of generation 5 was carried out using RT-qPCR.

Seventeen PEDV-positive samples were collected from farms affected by PEDV in the Hubei, Guangxi, and Jiangxi provinces. These samples were all confirmed to be positive for PEDV using quadruple RT-PCR, while TGEV, PDCoV, and PoRV tested negative. Virus isolation was performed in Vero cells following established protocols. After 4–5 passages, characteristic cytopathic effects of PEDV, primarily syncytia formed through cell fusion, were observed (Figure 1A). An immunofluorescence assay (IFA) was then conducted in Vero cells, revealing specific green fluorescence at the sites of cytopathic changes (Figure 1B). RT-qPCR further confirmed the expression of the PEDV N gene; the cycle threshold (CT) values are 8.21, 15.3, and 14.08, respectively (Figure 1C). Based on the collection sites and dates, the strains were named 2021-HBMC, 2024-JXNC, and 2024-JXYX and uploaded to the GenBank database (Genbank: PV235413-PV235415).

### 3.2. Neutralization Titer of AJ1102 Immune Serum Against Isolated Strains Is Significantly Reduced

**Figure 2:**
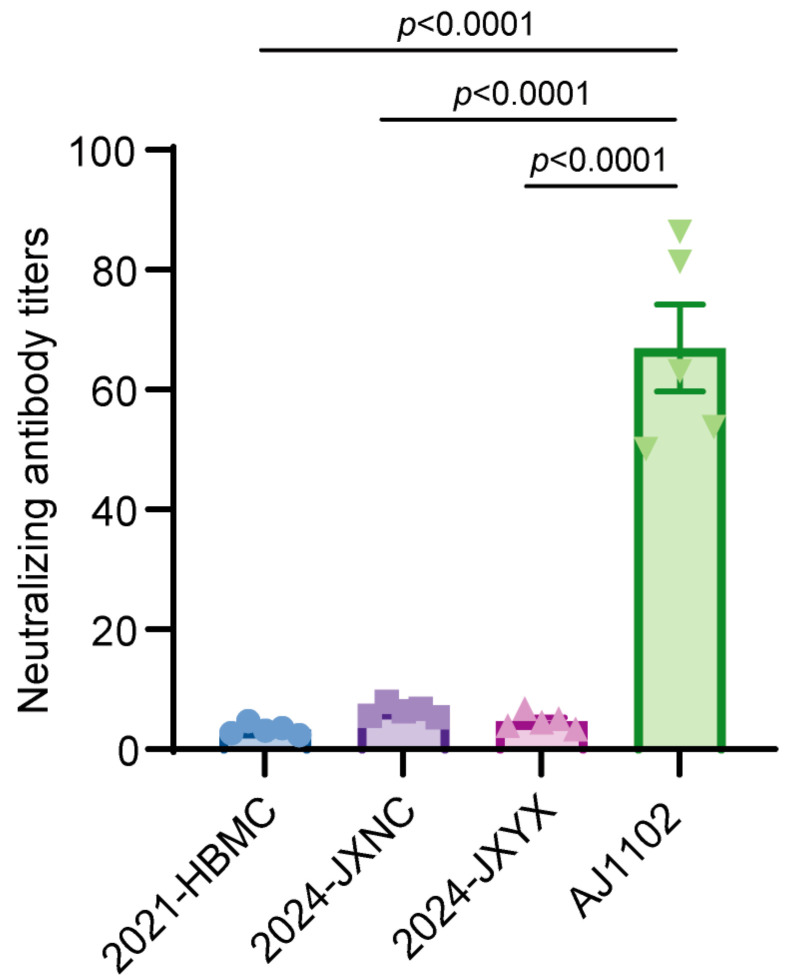
The neutralization titer of G2b immune serum against 2021-HBMC, 2024-JXNC, and 2024-JXYX strains. All values are expressed as the mean ± standard error of the mean (SEM). The data were subjected to one-way analysis of variance (ANOVA), and Dunnett’s multiple comparisons test was used to compare each group with the AJ1102 group using GraphPad Prism 9.5.1 software.

To investigate the cause of immunization failure, we conducted neutralization assays using immune sera from the G2b vaccine against isolated strains. The results revealed that the neutralization titers of the vaccine serum against the 2021-HBMC, 2024-JXNC, and 2024-JXYX strains were significantly lower than the titers observed for the AJ1102 reference strain. These findings suggest that the isolated strains in this study may exhibit antigenic variation (Figure 2).

### 3.3. Identity and Phylogenetic Analysis of the S Gene

**Figure 3:**
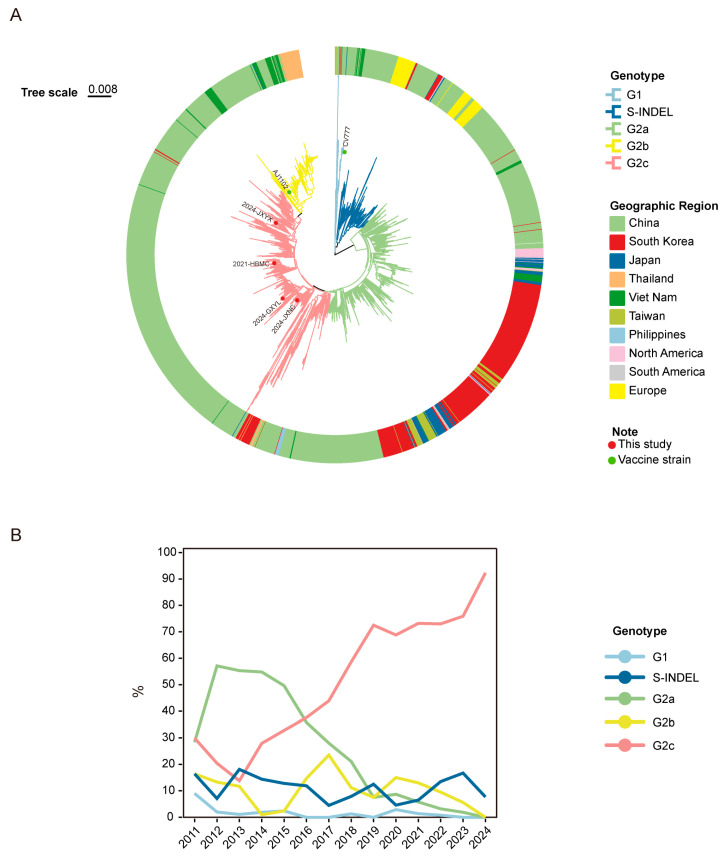
Genotyping of the PEDV strains based on S gene sequences from 2010 to 2024. (**A**) The S gene phylogenetic tree was generated by Mega7.0 using the Neighbor-Joining method with 1000 bootstrap replicates. The evolutionary branches of different genotypes are colored, and isolation countries are shown by different color blocks of leaves. (**B**) The isolation rate of each genotype in China from 2011 to 2024 was counted and is represented by line graphs.

To identify and analyze the genotype and genetic variation in endemic strains in these disease fields, we amplified and sequenced the S gene and constructed an S gene-based phylogenetic tree using the sequences of 29 representative PEDV strains and complete S gene sequences available in the NCBI database from 2010 to 2024 (Table S2). As shown in Figure 3A, PEDV is primarily classified into three types: G1, G2, and S-INDEL. The G2 type is further subdivided into G2a, G2b, and G2c subtypes. The strains identified in this study all belong to the G2c subtype. Specifically, 2021-HBMC, 2024-GXYL, 2024-JXNC, and 2024-JXYX are located in four distinct branches within G2c. Notably, 2021-HBMC is most closely related to the 2020 strain CH/SDZH/07/2020 (MZ161042), while 2024-JXNC is most closely related to the 2017 strain FY/2017 (MW826593). The strain 2024-GXYL is most closely related to the 2022 strain PEDV/CN/JSYC (PP901936), and 2024-JXYX is most closely related to the 2022 strain FJLY01-2022 (OP171894). These strains showed 92.1% to 93.2% similarity to the vaccine strain CV777 and 96.0% to 97.4% similarity to the vaccine strain AJ1102 (Table 1). Notably, the Chinese strains account for a significant proportion. We calculated the distribution of genotypes in China from 2011 to 2024. As depicted in Figure 3B, the detection rate of G2c strains has significantly increased since 2018, reaching 92.3% in 2024, while the detection rates of G2a and G2b strains have declined steadily. Additionally, all European strains (53/53) were exclusively classified as S-INDEL variants. In contrast, Korean and Japanese isolates predominantly clustered within the G2a genotype (89.03% and 91.78%, respectively), with minor representation of S-INDEL and G2c subtypes. Thai strains demonstrated strong G2b predominance (79.06%), showing closer phylogenetic proximity to the AJ1102 reference strain. Vietnamese strains exhibited the broadest genetic diversity: G2a (25.92%), G2b (51.8%), S-INDEL (11.11%), G2c (9.88%), and sporadic G1 variants. North American isolates primarily comprised the G2a-AH2012 lineage (76.2%), with residual S-INDEL populations (23.8%).

### 3.4. Comparative Analysis of Amino Acid Sequences of the Spike Protein

**Figure 4:**
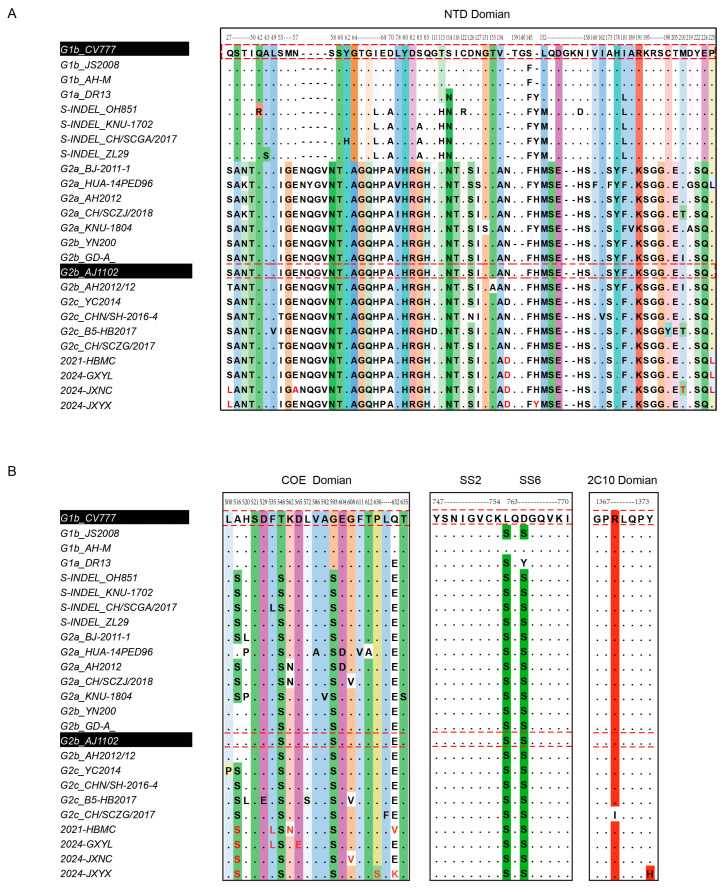
Amino acid variation in G2c strains isolated in this study in the S protein. The spike sequences of 2021-HBMC, 2024-GXYL, 2024-JXNC, and 2024-JXYX and the reference strains were aligned with Mega7.0, and the sites with variation were spiced using jalview 2.11.4.1. (**A**) Analysis of amino acid variations in the NTD region. (**B**) Analysis of amino acid variations in the COE, SS2, SS6 and 2C10 domain.

A comparative analysis of amino acid variations in the spike (S) protein, with a particular focus on the major neutralizing epitopes of PEDV, was conducted to understand the potential causes of vaccine-induced immune failure. As shown in Figure 4A, compared to the vaccine strain CV777, the strains 2021-HBMC, 2024-JXNC, 2024-JXYX, and 2024-GXYL exhibited mutations at multiple sites in the S protein. These strains shared characteristics with other G2-type variants, including consecutive insertions and deletions in the N-terminal domain (NTD) region. Additionally, compared to the vaccine strain AJ1102, mutations such as S27L, E57A, N139D, M214T, and P229L were observed. In the primary neutralizing epitope COE region, eight amino acid variations were identified relative to the AJ1102 strain, including A520S, F539L, K566N, D569E, G612V, P634S, and E636V/K. In the SS6 region, two amino acid mutations (L763S, D765S) were identified compared to CV777, and one mutation (Y1376H) was observed in the 2C10 region relative to AJ1102 (Figure 4B). Furthermore, the N-glycosylation sites of the isolated strains were analyzed. The 2021-HBMC, 2024-JXNC, 2024-JXYX, and 2024-GXYL strains exhibited consistent N-glycosylation sites with the G2c reference strain but lacked several N-glycosylation sites compared to CV777 (112NTSA, 127NKTL, 510NITV, 552NVTN), and compared to AJ1102, they were missing the 510NITV site. Additionally, the 2024-GXYL strain lacked the 347NSSD glycosylation site (Table 2).

### 3.5. Comparative Analysis of the Complete Genome of the 2021-HBMC Strain

**Figure 5:**
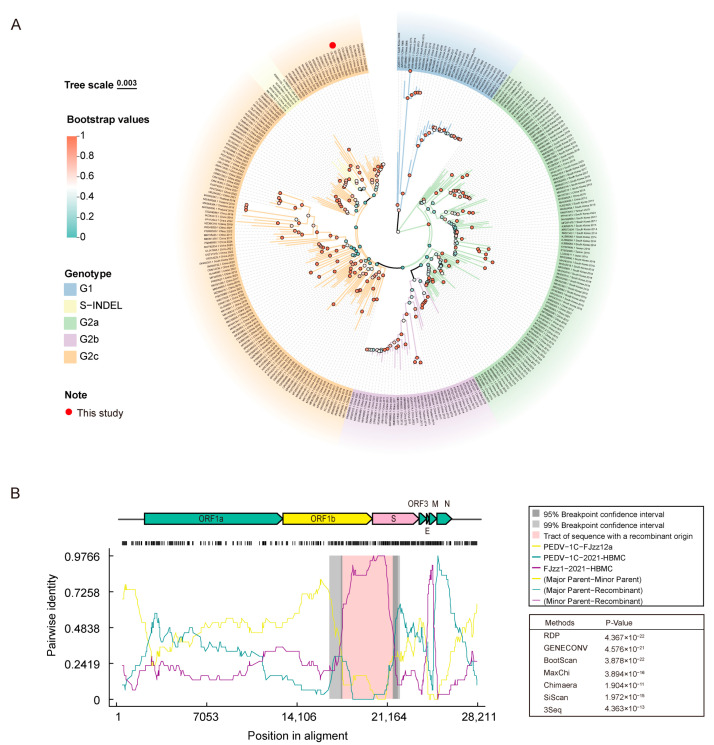
Phylogenetic and recombination analyses of full-genome sequences of 2021-HBMC and 290 reference strains. (**A**) The whole-genome phylogenetic tree was generated by Mega 7.0 using the maximum likelihood method with 1000 bootstrap replicates. The evolutionary genotypes are marked with different color blocks of leaves. And the information for the reference strains, such as accession number, name, isolation year, and countries, is marked on the leaves’ names. (**B**) Genome recombination analysis of 2021-HBMC was conducted using RDP4 with seven methods under default settings; recombination events were considered reliable if at least six methods identified them as positive and the *p*-value was below 10^−6^. The recombination region is marked with a pink box. The identity between the major parent and 2021-HBMC is represented by a green line, the identity between the minor parent and 2021-HBMC is represented by a purple line, and the identity between the major parent and minor parent is represented by a yellow line.

Whole-genome sequencing of the 2021-HBMC strain was performed, followed by genetic evolution and recombination analysis with 290 reference strains (Table S3). The results indicated that 2021-HBMC belongs to the G2c genotype, sharing the closest genetic distance with PEDV TRS2021 (OL762461). Both strains are placed in the same small branch, with a genetic similarity of 99.4% (Figure 5A). Recombination analysis revealed that the genomic regions ORF1b (4975–7962) and the S gene (1–914) of 2021-HBMC are likely the result of recombination between an unknown primary parent and the secondary parent FJzz1 (Figure 5B). This finding was supported by seven detection algorithms, all of which yielded *p*-values < 10^−6^.

## 4. Discussion

The porcine epidemic diarrhea (PED) epidemic has been widely reported in Asia, Europe, and North America since 2010, significantly impacting global swine production and attracting considerable attention [17]. According to statistics, PEDV infection was reported in almost all provinces of China from 2010 to 2020 [13]. Meanwhile, the high variability of PEDV has led to genetic diversity, posing challenges to vaccine efficacy. To better control PEDV infections, enhanced surveillance of prevalent PEDV strains is necessary, with next-generation sequencing (NGS) technology playing a critical role. However, the current PEDV genotyping standards are not yet standardized. These discrepancies primarily arise in the classification of G2 variant strains, mainly due to the diversity of the variant strains and the selection of reference strains [31]. Additionally, there is a significant issue regarding the diverse nomenclature for recombinant S-indel strains [16,26]. Additionally, the G2c strain is a recently defined subtype in several studies, previously included under G2a or G2b, but gradually evolving into a distinct branch, primarily composed of Chinese strains, and these strains have been responsible for PEDV infections in multiple regions of China [26,27,28]. Specifically, G2c is regarded as the major circulating subtype in East, North, South, and Central China between 2017 and 2021 [26]. From 2020 to 2022, the prevalence of G2c in Sichuan Province, China, reached as high as 87.28% [32]. From 2023 to 2024, G2c has continued to be the primary circulating subtype in Southwest China [33]. In conclusion, G2c has become the predominant circulating subtype in various regions of China. The spread of PEDV in China may be facilitated by the inter-provincial live pig trade, with Guangdong and Henan identified as key transmission hubs, and Hubei Province potentially serving as a third hub facilitating the spread to the southwest region [8]. However, no reports of the G2c strain have yet been documented in other countries.

To further investigate the genetic evolution of PEDV, this study conducted a genetic evolution analysis of PEDV S gene sequences from the NCBI database starting from 2010, using genotyping methods similar to Wan Liang et al., Yingshuo Sun et. al., and Cheng Yang et al. [27,28,34]. And the genotype distribution of Chinese strains was subsequently analyzed and summarized. The results showed that G2a strains dominated from 2010 to 2013, but their prevalence has steadily declined in the following years. In contrast, the number of G2c strains has increased consistently since 2018, reaching 92.3% by 2024. This trend indicates that G2c has become the predominant circulating strain in China, which aligns with recent research reports [26,27]. Therefore, it can be inferred that while current vaccines effectively control infections by some G2a and G2b strains, their efficacy against G2c PEDV may be limited. Additionally, although G2c strains were not previously reported in other countries, our analysis revealed that certain Korean strains (such as ON263454, OR125556) and Vietnamese strains (such as MK435380.1, MK435392.1) were also classified as G2c in the phylogenetic tree of the S gene. This suggests that the spread of G2c may not be limited to China alone, and the risk of its cross-border dissemination warrants further monitoring.

To investigate the cause of vaccine failure, this study collected piglet clinical samples from farms in Hubei, Jiangxi, and Guangxi provinces with immune failures (the pigs had been vaccinated with a commercial vaccine based on the G2b strain). Four PEDV strains were identified, and three epidemic strains were successfully isolated. Genetic evolution analysis revealed that all identified and isolated strains belonged to the G2c genotype. Notably, the neutralizing activity of the G2b vaccine immune serum was significantly reduced against these three G2c strains, suggesting that the neutralizing epitopes of the G2c strains may have undergone substantial variation. The S gene is the most mutation-prone region in the PEDV genome, and mutations in this region may alter the antigenicity, pathogenicity, and neutralizing properties of the S protein [7,23,35]. This study found that the S protein of the four G2c strains exhibited only 92.1–93.2% similarity to the CV777 strain and 96.0–97.4% similarity to the AJ1102 vaccine strain. Further analysis revealed several mutations in the key neutralizing epitopes (such as S-NTD, COE, SS6, and 2C10) of these strains compared to the AJ1102 vaccine strain, including the S-NTD epitopes S27L, E57A, N139D, M214T, and P229L; the COE epitopes A520S, F539L, K566N, D569E, G612V, P634S, and E636V/K; and the 2C10 epitope Y1376H. Among them, N139D, P229L, and A520S may affect the phosphorylation level of the PEDV S protein [36]. These mutations may significantly alter the virus’s antigenicity and neutralizing characteristics, thus leading to vaccine failure. But further in vitro and in vivo experiments are required to validate the specific effect of these mutations on viral virulence and immune evasion.

In addition, changes in the N-glycosylation sites of the coronavirus S protein are regarded as critical factors influencing viral infectivity, pathogenicity, and immunogenicity [37,38,39,40]. Our analysis revealed that the four G2c strains lacked five glycosylation sites compared to the CV777 strain and lacked the 510NITV site compared to the AJ1102 strain. Notably, the 2024-GXYL strain additionally lacked the 347NSSD site. The loss of these glycosylation sites may further alter the virus’ antigenicity and neutralizing characteristics, thereby exacerbating the risk of vaccine failure.

Recombination events play an essential role in the evolution of PEDV, contributing to its genetic diversity and allowing newly emerged viruses to rapidly adapt to environmental changes, such as evading host immune responses or enhancing viral virulence [31,41,42]. In this study, whole-genome sequencing of the 2021-HBMC strain was performed, followed by genetic evolution analysis using 290 reference strains. The results showed that the 2021-HBMC strain belonged to the G2c genotype. Notably, some reference strains exhibited discordance in their genotypes when comparing the whole-genome phylogenetic tree with the S gene phylogenetic tree. For instance, the S-INDEL strain OH851 was included in the G2c branch of the whole-genome phylogenetic tree, which could be due to viral recombination events. Recombination analysis using RDP4 indicated that the 2021-HBMC strain might have originated from a recombination event, with the minor parent identified as FJzz1, although the major parent remains undetermined. Further genome-wide comparisons are required to clarify its origin. In addition, to address the rapid evolution of PEDV, future efforts should focus on enhancing surveillance of circulating strains, as well as the development of novel vaccines based on these newly identified variants and the optimization of immunization strategies to improve PEDV control and mitigate its impact on the swine industry.

## 5. Conclusions

In conclusion, this study successfully isolated three PEDV strains from Chinese farms experiencing vaccine failures, all of which belong to the G2c genotype. These strains exhibited multiple mutations in key neutralizing epitopes of the S protein, leading to a significant reduction in neutralizing titers of vaccine-induced immune sera, thus contributing to vaccine failure. Moreover, the loss of glycosylation sites and potential recombination events may further exacerbate the antigenic variation of the virus. The findings of this study highlight the challenge posed by ongoing genetic variations of PEDV on vaccine efficacy and provide valuable insights for future vaccine development and control strategies.

## Figures and Tables

**Table 1:** Similarity between G2c strains identified in this study and vaccine strains.

PEDV Strains	CV777	AJ1102
2021-HBMC	93.1%	97.2%
2024-GXYL	92.1%	96.0%
2024-JXNC	93.2%	97.2%
2024-JXYX	93.0%	97.4%

**Table 2:** Variation in highly specific N-glycosylation sites in the spike protein of different genotypes.

Genotype	Strains	High-Specificity N-Glycosylation Sites														
		57	62	112	118	127	212	300	320	347	380	510	552	777	1245	1258
G1b	KT323979-CV777	–	–	NTSA	–	NKTL	NVTS	–	–	NSSD	–	NITV	NVTN	NISI	NKTL	–
G1b	KC109141-JS2008	–	–	NTSA	–	NKTL	NVTS	–	–	NSSD	–	NITV	NVTN	NISI	NKTL	–
G1a	JQ023161-DR13	–	–	–	–	NKTL	NVTS	–	NDTS	NSSD	–	NITV	NVTN	NISI	NKTL	NRTG
S-INDEL	KU847996-ZL29	–	–	–	–	NKTL	NVTS	–	NDTS	NSSD	–	–	–	NISI	NKTL	NRTG
S-INDEL	KJ399978-OH851	–	–	–	–	NKTL	NVTS	–	NDTS	NSSD	–	–	–	NISI	NKTL	NRTG
S-INDEL	MH243316-KNU-1702	NSSS	–	–	–	NKTL	NVTS	–	NDTS	NSSD	–	–	–	NISI	NKTL	NRTG
G2b	JX188454-AJ1102	–	NSTW	–	NATA	–	NVTS	–	NDTS	NSSD	–	NITV	–	NISI	NKTL	NRTG
G2b	KU646831-AH2012_12	–	NSTW	–	NATA	–	NVTS	–	NDTS	NSSD	–	NITV	–	NISI	NKTL	NRTG
G2a	JN825712-BJ-2011-1	–	NSTW	–	NATA	–	NVTS	–	NDTS	NSSD	–	–	–	NISI	NKTL	NRTG
G2a	KC210145-AH2012	–	NSTW	–	NATA	–	NVTS	–	NDTS	NSSN	–	–	–	NISI	NKTL	NRTG
G2a	MH061342-CH_SCZJ_2018	–	NSTW	–	NATA	–	NVTS	NKTI	NDTS	NSSD	–	–	–	NISI	NKTL	NRTG
G2c	KU252649-YC2014	–	NSTW	–	NATA	–	NVTS	–	NDTS	NSSD	–	–	–	NISI	NKTL	NRTG
G2c	MH161337-CH_SCZG_2017	–	NSTW	–	NATA	–	NVTS	–	NDTS	NSSN	NSTV	–	–	NISI	NKTL	NRTG
G2c	2021-HBMC	–	NSTW	–	NATA	–	NVTS	–	NDTS	NSSD	–	–	–	NISI	NKTL	NRTG
G2c	2024-GXYL	–	NSTW	–	NATA	–	NVTS	–	NDTS	–	–	–	–	NISI	NKTL	NRTV
G2c	2024-JXNC	–	NSTW	–	NATA	–	NVTS	–	NDTS	NSSN	–	–	–	NISI	NKTL	NRTG

## Data Availability

All data analyzed during this study are available from the corresponding author on reasonable request.

## Supplementary Materials

The following supporting information can be downloaded at https://www.mdpi.com/article/10.3390/vetsci12050444/s1: Table S1: Primers used for S gene amplification; Table S2: PEDV strains used for phylogenetic analysis of the S gene; Table S3: PEDV strains used for phylogenetic analysis of the genome.

## References

[1] Yang S., Liu G., Savelkoul H.F.J., Jansen C.A., Li B. (2023). Mini-review: Microbiota have potential to prevent PEDV infection by improved intestinal barrier. Front. Immunol..

[2] Stevenson G.W., Hoang H., Schwartz K.J., Burrough E.R., Sun D., Madson D., Cooper V.L., Pillatzki A., Gauger P., Schmitt B.J. (2013). Emergence of Porcine epidemic diarrhea virus in the United States: Clinical signs, lesions, and viral genomic sequences. J. Vet. Diagn. Investig..

[3] Li Q., Xu Z., Wu T., Peng O., Huang L., Zhang Y., Xue C., Wen Z., Zhou Q., Cao Y. (2018). A flagellin-adjuvanted PED subunit vaccine improved protective efficiency against PEDV variant challenge in pigs. Vaccine.

[4] Shibata I., Tsuda T., Mori M., Ono M., Sueyoshi M., Uruno K. (2000). Isolation of porcine epidemic diarrhea virus in porcine cell cultures and experimental infection of pigs of different ages. Vet. Microbiol..

[5] Wang Q., Vlasova A.N., Kenney S.P., Saif L.J. (2019). Emerging and re-emerging coronaviruses in pigs. Curr. Opin. Virol..

[6] Song D., Zhou X., Peng Q., Chen Y., Zhang F., Huang T., Zhang T., Li A., Huang D., Wu Q. (2015). Newly Emerged Porcine Deltacoronavirus Associated With Diarrhoea in Swine in China: Identification, Prevalence and Full-Length Genome Sequence Analysis. Transbound. Emerg. Dis..

[7] Lin C.M., Saif L.J., Marthaler D., Wang Q. (2016). Evolution, antigenicity and pathogenicity of global porcine epidemic diarrhea virus strains. Virus Res..

[8] He W.-T., Bollen N., Xu Y., Zhao J., Dellicour S., Yan Z., Gong W., Zhang C., Zhang L., Lu M. (2022). Phylogeography Reveals Association between Swine Trade and the Spread of Porcine Epidemic Diarrhea Virus in China and across the World. Mol. Biol. Evol..

[9] Kocherhans R., Bridgen A., Ackermann M., Tobler K. (2001). Completion of the porcine epidemic diarrhoea coronavirus (PEDV) genome sequence. Virus Genes.

[10] Huang Y.-W., Dickerman A.W., Piñeyro P., Li L., Fang L., Kiehne R., Opriessnig T., Meng X.-J. (2013). Origin, evolution, and genotyping of emergent porcine epidemic diarrhea virus strains in the United States. mBio.

[11] Wood E.N. (1977). An apparently new syndrome of porcine epidemic diarrhoea. Vet. Rec..

[12] Wang D., Fang L., Xiao S. (2016). Porcine epidemic diarrhea in China. Virus Res..

[13] Park J.E. (2024). Porcine Epidemic Diarrhea: Insights and Progress on Vaccines. Vaccines.

[14] Li W., Li H., Liu Y., Pan Y., Deng F., Song Y., Tang X., He Q. (2012). New variants of porcine epidemic diarrhea virus, China, 2011. Emerg. Infect Dis..

[15] Chen J., Liu X., Shi D., Shi H., Zhang X., Li C., Chi Y., Feng L. (2013). Detection and molecular diversity of spike gene of porcine epidemic diarrhea virus in China. Viruses.

[16] Guo J., Fang L., Ye X., Chen J., Xu S., Zhu X., Miao Y., Wang D., Xiao S. (2019). Evolutionary and genotypic analyses of global porcine epidemic diarrhea virus strains. Transbound. Emerg. Dis..

[17] Lee C. (2015). Porcine epidemic diarrhea virus: An emerging and re-emerging epizootic swine virus. Virol. J..

[18] Wang L., Byrum B., Zhang Y. (2014). New variant of porcine epidemic diarrhea virus, United States, 2014. Emerg. Infect. Dis..

[19] Li W., van Kuppeveld F.J.M., He Q., Rottier P.J.M., Bosch B.J. (2016). Cellular entry of the porcine epidemic diarrhea virus. Virus Res..

[20] Hou Y., Lin C.-M., Yokoyama M., Yount B.L., Marthaler D., Douglas A.L., Ghimire S., Qin Y., Baric R.S., Saif L.J. (2017). Deletion of a 197-Amino-Acid Region in the N-Terminal Domain of Spike Protein Attenuates Porcine Epidemic Diarrhea Virus in Piglets. J. Virol..

[21] Li Z., Ma Z., Dong L., Yang T., Li Y., Jiao D., Han W., Zheng H., Xiao S. (2022). Molecular Mechanism of Porcine Epidemic Diarrhea Virus Cell Tropism. mBio.

[22] Wen Z., Li J., Zhang Y., Zhou Q., Gong L., Xue C., Cao Y. (2018). Genetic epidemiology of porcine epidemic diarrhoea virus circulating in China in 2012–2017 based on spike gene. Transbound. Emerg. Dis..

[23] Su M., Li C., Qi S., Yang D., Jiang N., Yin B., Guo D., Kong F., Yuan D., Feng L. (2020). A molecular epidemiological investigation of PEDV in China: Characterization of co-infection and genetic diversity of S1-based genes. Transbound. Emerg. Dis..

[24] Bi J., Zeng S., Xiao S., Chen H., Fang L. (2012). Complete genome sequence of porcine epidemic diarrhea virus strain AJ1102 isolated from a suckling piglet with acute diarrhea in China. J. Virol..

[25] Won H., Lim J., Noh Y.H., Yoon I., Yoo H.S. (2020). Efficacy of Porcine Epidemic Diarrhea Vaccines: A Systematic Review and Meta-Analysis. Vaccines.

[26] Li X., Li Y., Huang J., Yao Y., Zhao W., Zhang Y., Qing J., Ren J., Yan Z., Wang Z. (2022). Isolation and oral immunogenicity assessment of porcine epidemic diarrhea virus NH-TA2020 strain: One of the predominant strains circulating in China from 2017 to 2021. Virol. Sin..

[27] Yang C., Sun J.-Y., Li X.L., Cheng N., Wang K.-Y., Li L.-Q., Cheng X.-J., Sun Y.-F. (2024). Emerging and re-emerging genotype 2c porcine epidemic diarrhoea virus with high pathogenicity in China. J. Infect..

[28] Sun Y., Gong T., Wu D., Feng Y., Gao Q., Xing J., Zheng X., Song Z., Liu X., Chen X. (2023). Isolation, identification, and pathogenicity of porcine epidemic diarrhea virus. Front. Microbiol..

[29] Xie J., Chen Y., Cai G., Cai R., Hu Z., Wang H. (2023). Tree Visualization By One Table (tvBOT): A web application for visualizing, modifying and annotating phylogenetic trees. Nucleic Acids Res..

[30] Yu J., Chai X., Cheng Y., Xing G., Liao A., Du L., Wang Y., Lei J., Gu J., Zhou J. (2018). Molecular characteristics of the spike gene of porcine epidemic diarrhoea virus strains in Eastern China in 2016. Virus Res..

[31] Tian Y., Yang X., Li H., Ma B., Guan R., Yang J., Chen D., Han X., Zhou L., Song Z. (2021). Molecular characterization of porcine epidemic diarrhea virus associated with outbreaks in southwest China during 2014–2018. Transbound. Emerg. Dis..

[32] Peng Q., Fu P., Zhou Y., Lang Y., Zhao S., Wen Y., Wang Y., Wu R., Zhao Q., Du S. (2024). Phylogenetic Analysis of Porcine Epidemic Diarrhea Virus (PEDV) during 2020-2022 and Isolation of a Variant Recombinant PEDV Strain. Int. J. Mol. Sci..

[33] Xie B., Yan W., Yang X., Fan H. (2025). Molecular characterization of porcine epidemic diarrhea virus in Sichuan from 2023 to 2024. Microb. Pathog..

[34] Liang W., Zhou D., Geng C., Yang K., Duan Z., Guo R., Liu W., Yuan F., Liu Z., Gao T. (2020). Isolation and evolutionary analyses of porcine epidemic diarrhea virus in Asia. PeerJ.

[35] Su M., Wang Y., Yan J., Xu X., Zheng H., Cheng J., Du X., Liu Y., Ying J., Zhao Y. (2024). Isolation and characterization of a novel S1-gene insertion porcine epidemic diarrhea virus with low pathogenicity in newborn piglets. Virulence.

[36] Sun J., Cheng J., Shi D., Xu X., Liu Y., Ying J., Zhao Y., Zheng H., Yan J., Sun D. (2025). Genetic Epidemiology of Porcine Epidemic Diarrhea Virus Circulating in China From 2010 to 2024, Characterization of Phylogenetic and Genetic Diversity of S1-Based Genes. J. Med. Virol..

[37] Le B.T., Gallage H.C., Kim M.-H., Park J.-E. (2023). Molecular Characterization of Porcine Epidemic Diarrhea Virus from Field Samples in South Korea. Viruses.

[38] Zhu H., Lou J., Yang Z., Bai J., Jiang P., Wang X., Liu X. (2025). STT3B promotes porcine epidemic diarrhea virus replication by regulating N-glycosylation of PEDV S protein. J. Virol..

[39] Huang H.-C., Lai Y.-J., Liao C.-C., Wang F.-Y., Huang K.-B., Lee I.-J., Chou W.-C., Wang S.-H., Wang L.-H., Hsu J.-M. (2021). Targeting conserved N-glycosylation blocks SARS-CoV-2 variant infection in vitro. EBioMedicine.

[40] Aloor A., Aradhya R., Venugopal P., Gopalakrishnan Nair B., Suravajhala R. (2022). Glycosylation in SARS-CoV-2 variants: A path to infection and recovery. Biochem. Pharmacol..

[41] Wang Y., Liu D., Shi W., Lu R., Wang W., Zhao Y., Deng Y., Zhou W., Ren H., Wu J. (2015). Origin and Possible Genetic Recombination of the Middle East Respiratory Syndrome Coronavirus from the First Imported Case in China: Phylogenetics and Coalescence Analysis. mBio.

[42] Liu H., Yin X., Tian H., Qiu Y., Wang Z., Chen J., Ma D., Zhao B., Du Q., Tong D. (2022). The S protein of a novel recombinant PEDV strain promotes the infectivity and pathogenicity of PEDV in mid-west China. Transbound. Emerg. Dis..

